# Functionally distinct core microbes of *Tricholoma matsutake* revealed by cross-study analysis

**DOI:** 10.1186/s40168-025-02329-x

**Published:** 2026-02-04

**Authors:** Shinnam Yoo, Chang Wan Seo, Young Woon Lim

**Affiliations:** School of Biological Sciences and Institute of Biodiversity, Seoul National University, Seoul, 08826 Republic of Korea

## Abstract

**Background:**

*Tricholoma matsutake* (TM), a prized wild mushroom in Eurasia, hosts distinct microbiomes in its mycorrhizal zone (shiro), with some microbes known to benefit TM. However, no study has systematically compared shiro-inhabiting microbiomes across multiple studies from either taxonomic or functional perspectives.

**Results:**

We first compiled bacterial and fungal amplicon sequences from public and newly generated datasets, then applied phylogenetic tree-based clustering to integrate technically heterogeneous sequences. This enabled the identification of core microbial phylotypes conserved in shiro from geographically diverse regions. We also revealed niche-specific phylotypes within the shiro, distinguishing those associated with soil, TM-colonized root, and fruitbody, thereby demonstrating clear niche differentiation. Functional predictions and experimental validation highlighted key roles of the microbes in degradation of aromatic compounds, utilization of plant-derived compounds, and fruitbody development.

**Conclusions:**

Our cross-study integration of shiro microbial sequences identified core and niche-specific phylotypes with distinct ecological roles. This study lays a foundation for advancing ecological research and cultivation strategies for TM.

**Supplementary Information:**

The online version contains supplementary material available at 10.1186/s40168-025-02329-x.

## Background

*Tricholoma matsutake* (TM) is a highly valued wild mushroom. The global retail market for matsutake (TM and related species) is estimated at $500 million, with wholesale prices ranging from $30 to $1,000/kg [1]. TM forms an ectomycorrhizal (EcM) symbiosis with the roots of host trees. It is found in the Northern Hemisphere, under Pinaceae in Asia (Bhutan, China, Japan, and Korean Peninsula) and Europe (Finland, Sweden, and Ukraine), but also under Fagaceae in Bhutan and Southwest China [2, 3]. Many residents near TM habitats rely heavily on harvesting and trading this species for their livelihood [4]. However, TM populations face sustainability challenges due to climate change [5, 6], host diseases [7, 8], deforestation [9], and overharvesting [10, 11], eventually being listed as an endangered species [12, 13].

Given the challenges in sustaining wild TM populations, interaction between TM and other microbes has been one of the primary focuses of research to cultivate this species. In “shiro”, a mycorrhizal zone of TM, a unique microbial community is structured by TM [14–19]. This phenomenon has drawn attention to the potential role of shiro microbes in supporting TM growth. Since the pioneering studies on shiro bacteria [14] and fungi [18], researchers have identified distinct microbial taxa distinguishing shiro soil from non-shiro soil [15, 20–28]. In addition, shiro microbes have been detected from various niches, including roots [29–36], fruitbodies [37–41], and even leaves and branches of host plants colonized by TM [33]. Some of these microbes have shown to benefit TM by promoting mycelial growth [22, 23, 31, 32, 40], detoxifying tolaasin produced by fruitbody-pathogen [42], and suppressing the growth of molds inhabiting the fruitbody [40].

Although metabarcoding (i.e., Next Generation Sequencing) and barcoding (i.e., Sanger sequencing) approaches have identified numerous shiro microbes, our understanding of these microbes remains fragmented due to taxonomic inconsistencies among the studies [3]. These inconsistencies stem from heterogeneity in sequencing platforms, target sequencing regions, reference databases, and taxonomic assignment tools, further aggravated by reporting results at varying taxonomic levels. To overcome taxonomic fragmentation, cross-study comparisons are essential to identify core microbial phylotypes conserved across habitats, which play key roles in ecosystem function [43]. In addition, investigating functional profiles (e.g., metabolic pathways and enzymatic reactions) may provide complementary insights, especially when taxonomically distinct microbiomes perform similar functions owing to functional redundancy [44]. However, no global comparison of shiro microbes has yet been conducted either from a taxonomic or functional perspective.

Our primary goal was to identify shiro core microbial phylotypes globally conserved across shiro, as well as niche-specific phylotypes within shiro uniquely associated with soil (S-soil), TM-colonized roots in shiro (S-root), and fruitbodies (S-fruitbody). To achieve this, we collected all publicly available microbial amplicon sequences from shiro, including some newly generated sequences from this study. These sequences were then integrated using a phylogenetic tree-based clustering approach. Building on this, we investigated the functional profiles of shiro through community-level functional prediction and phylotype-level experimental validation. Our findings on shiro core microbial phylotypes and their functions provide a foundation for future ecological research on shiro microbes.

## Methods

### Collection of studies

Studies were collected using the following search term on Google Scholar on 2024-09-25: "matsutake" AND ((("bacteria" OR "bacterial") AND ("16S" OR "rRNA" OR "rDNA" OR "SSU" OR "small subunit")) OR (("fungi" OR "fungal") AND ("ITS*region" OR "ITS1" OR "ITS2" OR "5.8S" OR "internal transcribed spacer" OR "rRNA" OR "rDNA"))). The collected studies were filtered using the following criteria: (1) “matsutake” is included in the title or abstract, (2) title and abstract are written in English, (3) focused on direct investigation of bacteria or fungi from natural shiro, (4) 16S rRNA gene (for bacteria) or ITS (for fungi) metabarcoding or barcoding (e.g. Sanger sequencing) sequence data were available in online databases such as GenBank or the Sequence Read Archive (SRA). In the final dataset, we included unpublished SRA metabarcoding data found using the search term "matsutake", along with newly generated metabarcoding and barcoding data from this study. Sampling locations were mapped using the rworldmap [45] package in R, based on GPS coordinates or, when unavailable, county-level coordinates extracted from SRA metadata or articles.

### Sampling and sequencing of microbes

We newly generated metabarcoding and barcoding data from shiro samples collected from South Korea between September 2020 and April and May 2021 (Table S1). The collected samples were divided into S-soil and S-roots (S-root tips for bacteria). The presence of TM in the shiro was verified using TM-specific primers (DTmF, DTmR) under the conditions described in a previous study [46]. Bacterial and fungal communities were investigated using both metabarcoding and barcoding approaches. For metabarcoding, the bacterial community was investigated from S-soil and S-root tips (V1–V4, PacBio Sequel), while the fungal community was investigated from S-roots (ITS2, Illumina MiSeq). For barcoding analysis, bacterial and fungal strains were isolated using solid media, and communities were analyzed using the V1V4 region of the 16S rRNA gene for bacteria and the full ITS region for fungi. For a detailed description of sampling, isolation, and sequencing strategy, see Supplementary Methods.

### Processing sequence data

Amplicon sequence data were processed according to their sequencing method (metabarcoding or barcoding). Our workflow for sequence analysis included data preprocessing, filtering, trimming, database construction, phylogenetic placement, tree decomposition and refinement, clustering, followed by phylotyping and taxonomic assignment (Fig. S1). Metabarcoding data were preprocessed for each study using QIIME2 v2023.9.1. Primers were trimmed using cutadapt v4.5 (--p-error-rate = 0.1), and the trimmed reads were denoised and clustered into amplicon sequence variants (ASVs) using DADA2 v1.26.0 (--p-trunc-len = 0, --p-max-ee = 8, --p-trunc-q = 2). Parameters were customized according to the sequencing platform, quality, and PCR primers (Table S2). ASVs were initially classified using the naïve lowest common ancestor (LCA) algorithm in MEGAN v6.21.1 [47] based on Nucleotide BLAST results. The BLAST search was conducted against the GenBank database (accessed on 25 October 2024) with max_target_seqs = 10 option. The initial LCA classification was performed with min percent identity = 70.0, top percent = 95.0, percent to cover = 51.0 options. Non-target ASVs were subsequently removed based on the results of the initial classification. Error-prone ends of the remaining ASVs were trimmed using the Reference_Blast_Extract function implemented in SuperCRUNCH v1.3.2 [48]. ASVs without matching sequences or shorter than 200 bp after trimming were discarded, along with singleton ASVs. The remaining ASVs were clustered with 100% identity using q2-vsearch v2023.9.0 [49]. Barcoding sequences were trimmed with SuperCRUNCH and sequences under 200 bp were removed. Finally, metabarcoding ASV sequences and barcoding sequences were combined as query sequences.

### Database construction

Databases were downloaded and modified for accurate identification of microbial sequences. The EzBioCloud database (accessed on 3 March 2022) [50] was used as the bacterial 16S database after removing archaeal sequences. The UNITE v9 dynamic database [51] and the fungal ITS RefSeq database (PRJNA177353) were modified by removing sequences labeled as “sp.” and those shorter than 300 bp. The two fungal ITS databases were then merged, with overlapping species removed based on accuracy of identity in the following order: UNITE representative sequences, UNITE reference sequences, and PRJNA177353. The combined database was used as the final fungal ITS database. Database sequences were aligned using WITCH v1.0.4 with the default option for each kingdom [52]. The resulting backbone alignment was used to infer a backbone tree with VeryFastTree [53], and branch lengths were optimized using RAxML-NG [54] on the fixed topology. The backbone trees of bacteria and fungi were rooted with monophyletic Fusobacteria and Rozellomycota, respectively.

### Phylogenetic placement, phylotyping, and taxonomic assignment

To incorporate query sequences into a unified phylogenetic framework, phylogenetic placement was performed. The query sequences were divided into chunks of 500 using Gappa [55], placed onto the backbone tree using App-SpaM with five fixed sets of binary patterns (*p* = 5) [56], and the resulting placements were merged with Gappa. Since the phylogenetic placement cannot resolve the relationship among the queries, the tree had to be refined. Therefore, uDance [57] was used to decompose and refine the tree through a divide-and-conquer approach. After decomposing the tree into chunks with 500 tips (which correspond to sequences) each, refinement was made with some modifications (Supplementary Methods).

To obtain generalizable taxonomic units, heterogeneous sequences were clustered into phylotypes and assigned taxonomy. The refined subtrees were midpoint-rooted using FastRoot [58], and tips were initially clustered using TreeCluster v1.0.4 (branch length threshold = 0.02, single linkage) [59]. Long-branched tips were identified with TreeShrink v1.3.9 [60]. Monophy [61] was used to verify monophyly of each cluster according to the following steps: (1) tips within a cluster were classified as monophyletic, non-monophyletic, or monotypic clades; (2) non-monophyletic clades were split into a major monophyletic clade and outliers; and (3) outliers were reassigned to unique clusters. This iterative process continued until no outliers were left, and the final clusters were defined as phylotypes. Phylotypes were then taxonomically assigned using a custom tree-walking algorithm: (1) If a phylotype contained both query and database tips, the query tips were assigned to the consensus taxonomy of the database tips. (2) If a phylotype contained only query tips, the algorithm walked up to the LCA, ignoring monotypic or long-branched database tips to avoid misassignment. Once valid database tips were found, the queries were assigned to their consensus taxonomy but above the species level. Each phylotype received a species name or LCA-based taxonomy with a unique ID in the format pt x.y or pt x.y.z, where x, y, and z represent the number of subtree, cluster, and subcluster. For monotypic phylotypes, “M” replaced y.

### Microbial diversity and taxonomic composition

For soil metabarcoding samples with missing metadata, we predicted the soil type based on TM read abundance. Using univariate clustering (Ckmeans.1d.dp [62]), the samples were assigned to either S-soil or non-shiro soil (NS-soil). When both bacterial and fungal samples were obtained from the same soil, the bacterial community was assigned the soil type of the corresponding fungal community. Depending on the analytical purpose, rarefaction was either applied or omitted: for alpha diversity and niche specificity analyses, rarefaction was applied. For these analyses, samples with fewer than 100 reads were removed in each study, and the remaining samples were rarefied to the lowest read count. In contrast, to retain S-soil samples with low read counts, rarefaction was omitted for taxonomic composition and beta diversity analyses (Fig. S2). Normalization, filtering, grouping, diversity, and statistical analysis were performed using the microeco package in R [63].

To compare alpha diversity between S-soil and NS-soil by each study, the Shannon index was calculated and compared using Kruskal–Wallis tests. Beta diversity was assessed using weighted UniFrac distances, calculated from an incremental phylogenetic tree generated via uDance [64]. Mantel tests were performed using the vegan [65] package to assess the correlation between phylogenetic dissimilarity and geographic distance, based on Pearson correlation with 999 permutations. Principal Coordinates Analysis (PCoA) and PERMANOVA were used to visualize and test beta diversity separation between S-soil and NS-soil samples within each study. Taxonomic profiles were visualized with heat trees using metacoder [66]. S-soil core phylotypes were defined as phylotypes consistently detected across all geographic regions, present in ≥ 50% of studies, and exhibiting ≥ 0.1% mean relative abundance across S-soil samples.

### Niche specificity analysis

To assess the niche specificity of microbial phylotypes, those with a mean relative abundance below 0.1% across all samples were excluded prior to analysis. Two complementary strategies were employed: (1) within-study comparisons between S-soil and NS-soil to ensure consistency among independent datasets, and (2) cross-study comparisons among niches within the shiro (S-soil, S-root tip, and S-fruitbody). In the first strategy, S-soil specificity of phylotypes was identified independently for each study that included both S-soil and NS-soil samples using three different analytical methods: LEfSe (LDA ≥ 3, *p* ≤ 0.05 with 1,000 bootstraps), Indicator Species Analysis (ISA, *p* ≤ 0.05 using 1,000 permutations via the r.g function in the indicspecies package [67]), and co-occurrence analysis using q2-SCNIC [68]. For co-occurrence analysis, phylotypes present in fewer than 20% of samples were excluded, and those positively correlated with TM (*r* ≥ 0.35) were considered significantly related to S-soil. A phylotype was considered S-soil-specific if it was statistically significant in at least one analytical method and was consistently significant in at least two independent studies. In the second strategy, niche specificity of S-root and S-fruitbody was assessed by comparing S-soil, S-root, and S-fruitbody samples using LEfSe (*p* ≤ 0.05 with 1,000 bootstraps, LDA ≥ 4 for bacteria and ≥ 3.5 for fungi). To identify interactions among niche-specific phylotypes within the S-fruitbody, a co-occurrence network analysis was performed on S-fruitbody samples. The resulting network was clustered using the Markov clustering algorithm implemented in clusterMaker2 [69] and visualized with Cytoscape [70].

### Functional prediction of microbial communities

Function of bacterial communities were predicted using PICRUSt2 v2.4.1 with default parameters [55, 71–74]. Bacterial functions were represented by MetaCyc pathways [75] or enzyme commission (EC) numbers. Function of fungal communities was predicted using FunFun, with e = 0.5 and K = 10 parameters [76]. FunFun utilizes 5,882 fungal genomes, offering a broader genomic basis compared to PICRUSt2, which uses 190 fungal genomes. Fungal functions were represented by KEGG orthology groups. To identify differentially enriched metabolic pathways and enzymatic reactions between niches, DESeq2 v1.44.0 was employed with default settings [77]. Functions with an adjusted *p*-value (Benjamini-Hochberg correction) ≤ 0.05 were considered significantly enriched.

S-soil-enriched functions were identified by comparing predicted expression levels between S-soil and NS-soil samples within each study and selecting functions significantly enriched in S-soil in at least two studies. Similarly, S-root- and S-fruitbody-enriched functions were identified by comparing each to the S-soil profile. In all comparisons, function tables were rarefied to the minimum total expression count. Enriched functions were clustered by predicted expression levels across samples using pvclust [78] with Canberra distance, average linkage, and 1,000 bootstraps. All heatmaps were generated using ComplexHeatmap [79].

### Experimental validation of bacterial functions

Experimental validation of the predicted functions was performed only for bacteria. To validate predicted bacterial community functions at the phylotype level, substrate utilization ability was investigated using Phenotype MicroArray™ (PM). Representative strains of the selected phylotypes were cultured on TSA media at 25°C for 3 days. Cells were collected, homogenized in 1x IF-0a solution by vortexing, adjusted to an optical density of 0.071 at 750 nm using a Genesys-5 spectrophotometer, and inoculated onto PM1 and PM2 plates. Plates were sealed with sterile CyclerSeal (Axygen, USA) and incubated at 25°C. After incubation, optical density was measured at 590 nm (OD590, respiration) and 750 nm (OD750, growth) every 24 hours using a FlexStation 3 microplate reader for eight days. PM wells exhibiting abiotic dye reduction were excluded from analysis. Growth curves were fitted using GP regression, and growth parameters (carrying capacity, area under the curve, and maximum growth rate) were calculated using AMiGA [80]. Growth parameters for all wells were normalized by subtracting the growth parameters of negative control wells (A1) for each PM plate. To compare utilization ability among strains, growth parameters were scaled from −100 to 100 for each strain, with parameters for A1 set to 0. Then, the activity index (AV) was calculated using k-means clustering [81], where 1 represents the lowest and 9 the highest utilization ability. Substrates were clustered by activity index across strains using pvclust.

## Results

### Collection of studies and sequence analysis

**Fig. 1:**
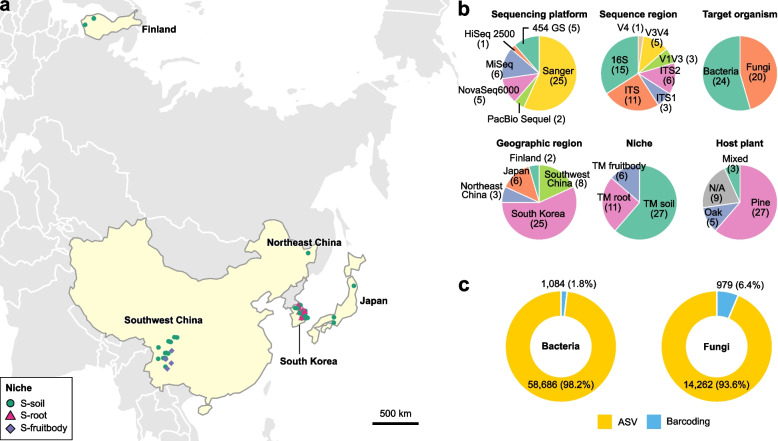
Global sequence dataset of shiro microbes. **a** Geographic distribution of sampling sites for public and newly generated sequence data across East Asia and Finland, with colors representing different niches of shiro: S-soil (green), S-root (magenta), and S-fruitbody (purple). GPS coordinates were used when available from SRA metadata or articles; otherwise, only locations identified with at least county-level resolution were mapped. Sampling sites for NS-soil samples are not shown as their coordinates overlapped with S-soil samples. **b** Summary of 44 datasets grouped by geographic region, niche, host plant, sequencing method, target organisms, and sequence region. **c** Donut charts depict the total number of sequences retrieved from bacterial and fungal datasets, with colors showing the proportions of metabarcoding ASVs (yellow) and barcoding sequences (blue)

Studies on shiro microbes were systematically collected from Google Scholar using targeted search terms. Among the collected studies, 25 studies met the aforementioned criteria. In addition to the datasets from 25 selected studies, two unpublished datasets (ST10 and ST15) from the SRA and newly generated datasets from this study (ST28) were also included. In total, datasets from 28 studies were subjected to the analysis (Table S2). These studies encompassed samples collected from Finland, Southwest China, Northeast China, South Korea, and Japan (Fig. 1a). From the collected studies, 44 datasets were defined based on sequencing platform, sequencing region, and target organism, and they encompassed diverse geographic regions, niches, and host plants (Fig. 1b). An additional 21 studies lacking sequence data, including key historical works, were reviewed (Table S3).

After processing the sequence datasets, we obtained 58,686 bacterial ASVs from 119 metabarcoding samples and 14,262 fungal ASVs from 193 metabarcoding samples. In addition, we obtained 1,084 bacterial and 979 fungal barcode sequences from Sanger sequencing, which included sequences obtained from cultured isolates and from culture-independent approaches (e.g., PCR-DGGE). In total, 59,770 bacterial and 15,241 fungal query sequences were obtained (Fig. 1c). The query sequences were placed into the backbone trees for clustering and taxonomic assignment, yielding 39,530 bacterial and 8,350 fungal phylotypes (Tables S4 and S5).

Soil metabarcoding samples with missing metadata were reclassified as S-soil or NS-soil. Fungal community samples were reclassified based on the relative abundance of TM, which averaged 62.1% ± 19.7% (SD) in S-soil samples and 2.0% ± 4.7% (SD) in NS-soil samples. Bacterial communities from the same environmental samples were classified accordingly. S-soil samples showed lower α-diversity than NS-soil samples in both bacterial and fungal communities (Fig. S3). S-soil samples also exhibited distinct microbial community compositions compared to NS-soil samples, based on weighted UniFrac distances (Fig. S4). As all 18 samples from ST23 were reclassified as NS-soil, the study was excluded, and 27 studies were subjected to further analysis.

### Comparison of shiro microbes across sequencing methods, geographic regions, and niches

**Fig. 2:**
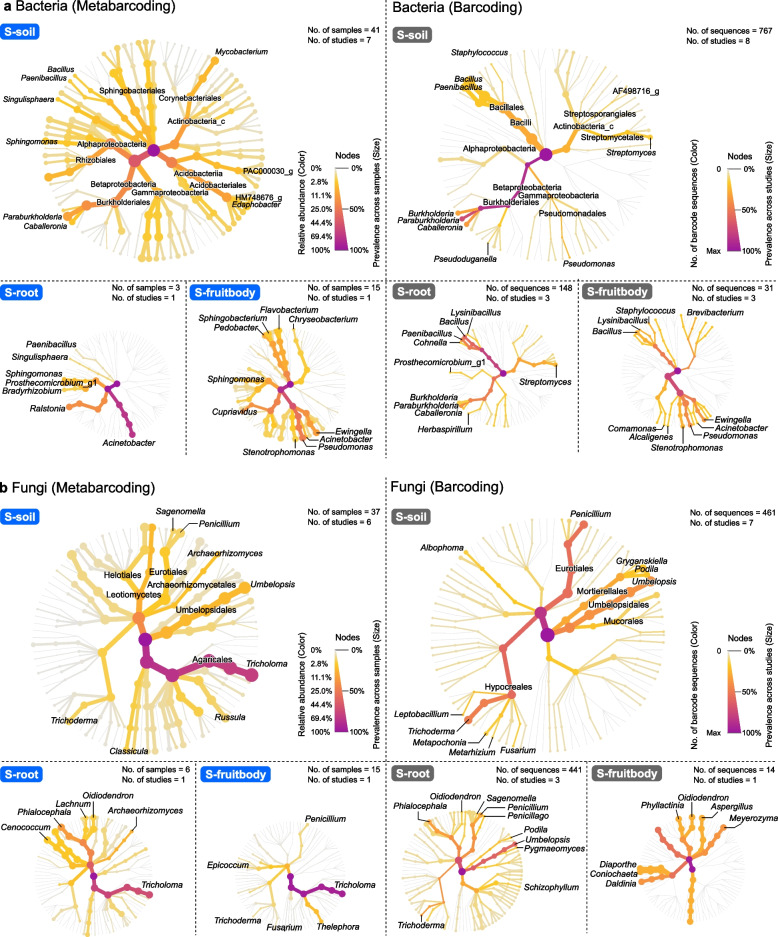
Shiro microbial taxa detected by different sequencing methods. Heat trees depict taxonomic profiles of bacteria (**a**) and fungi (**b**) across different niches, including S-soil, S-root, and S-fruitbody. Blue labels indicate metabarcoding data, and grey labels represent barcoding data. In metabarcoding data, node color (yellow to purple) represents relative abundance, and node size reflects prevalence across samples. In barcoding data, node color indicates the number of sequences, and node size reflects prevalence across studies. The number of samples, sequences, and studies is shown in each subpanel

Taxonomic comparison of metabarcoding and barcoding datasets revealed both overlaps and biases between sequencing methods (Fig. 2). Some taxa were abundantly detected from both sequencing methods. For bacteria, Betaproteobacteria (*Caballeronia* and *Paraburkholderia*) were commonly abundant in S-soil, while Gammaproteobacteria (*Ewingella* and *Pseudomonas*) were abundant in fruitbodies; among fungi, *Umbelopsis* was commonly abundant in S-soil, while Helotiales (*Phialocephala*, *Oidiodendron*, and *Lachnum*) were abundant in S-root. Despite these overlaps, notable taxonomic biases were observed. Barcoding tended to overrepresent *Bacillus*, *Paenibacillus*, and *Streptomyces* from S-soil and S-root. Among fungi, *Penicillium* and *Trichoderma* were similarly overrepresented in S-soil. In contrast, bacterial members of Alphaproteobacteria, Acidobacteriia, and *Mycobacterium*, as well as fungal taxa belonging to *Tricholoma*, *Archaeorhizomyces*, and Leotiomycetes were underrepresented from S-soil when investigated with barcoding.

**Fig. 3:**
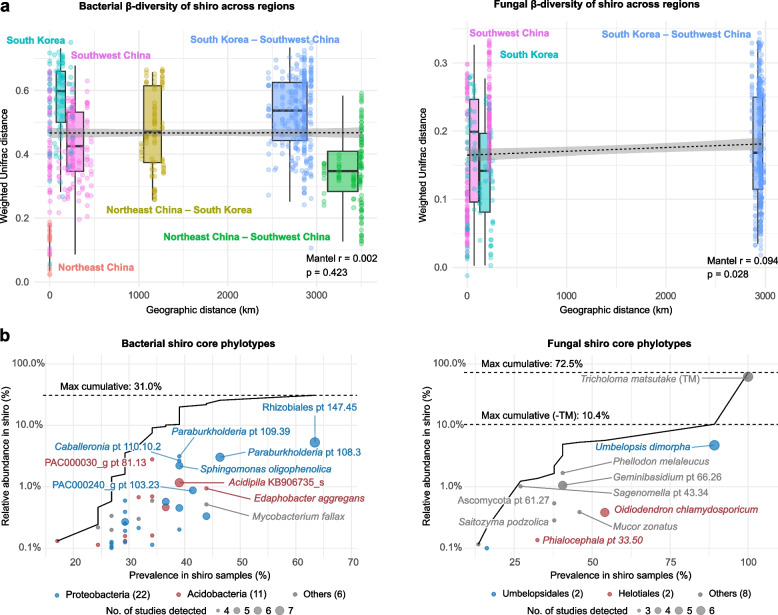
Comparison of shiro microbiome across geographic regions. **a** Mantel correlation between geographic distance and weighted UniFrac distance among shiro samples. Colored boxplots represent pairwise regional comparisons, and dots indicate individual comparisons. **b** Relative abundance and sample prevalence of core phylotypes across shiro samples. These phylotypes were identified from all geographic regions, with relative abundance ≥ 0.1% and study prevalence ≥ 50%. Circle size indicates study prevalence (the number of studies in which each phylotype was detected), and color represents taxonomic group. The number of core phylotypes for each taxonomic group is given in parentheses. Labeled phylotypes represent the top 10 phylotypes in terms of abundance and prevalence. Black line plot shows the cumulative relative abundance of the plotted phylotypes

To assess the biogeographic pattern of shiro microbiome, we examined their phylogenetic structure across regions. Phylogenetic structure varied both within and between geographic regions (Fig. 3a). However, this variation in bacterial communities was not correlated with geographic distance (*r* = 0.002, *p* = 0.42). For example, bacterial communities from Northeast China and Southwest China, collected over 3,500 km, were phylogenetically similar (mean UniFrac distance = 0.35), whereas samples from South Korea, collected over short distances, were highly dissimilar (mean UniFrac distance = 0.57). Fungal phylogenetic composition changed weakly with geographic distance (*r* = 0.094, *p* = 0.028).

Despite the variability of shiro microbiome, 39 bacterial and 12 fungal phylotypes were identified as shiro core phylotypes (Fig. 3b, Table S6). These were found in shiro across all studied regions, in at least half of the studies, and with a mean relative abundance ≥ 0.1%. Although they represented only 0.4% and 1.1% of the total number of bacterial and fungal phylotypes, respectively, they accounted for 31.0% and 72.5% (10.4% without TM) of the total community abundance, respectively. Most of the bacterial core phylotypes belonged to Proteobacteria (22 phylotypes) and Acidobacteria (11 phylotypes). Within Proteobacteria, Rhizobiales pt 147.45 (closely related to *Bradyrhizobium* AXAI_s) and *Paraburkholderia* pt 108.3 were the most abundant and prevalent. Among Acidobacteria, *Edaphobacter aggregans* and *Acidipila* KB906735_s were the most prevalent. In fungal communities, *Umbelopsis dimorpha* and *Oidiodendron chlamydosporicum* were the most prevalent, occurring in more than half of the shiro samples. However, shiro core phylotypes were also abundant in NS-soil, accounting for 25.0% of bacterial and 11.0% (without TM) of fungal abundance.

**Fig. 4:**
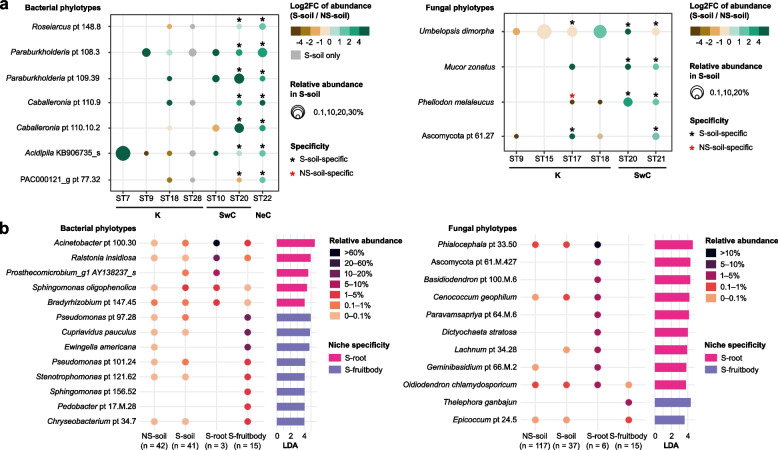
Niche-specificity of shiro microbial phylotypes. **a** Dot plots display S-soil-specific phylotypes, with dot color indicating the log2 fold-change of abundance between S-soil and NS-soil samples, and dot size indicating mean relative abundance in S-soil. Geographic regions for each study are represented by abbreviations: K = South Korea, SwC = southwestern China, NeC = northeastern China. Asterisks mark S-soil-specificity in the corresponding study, while red asterisks indicate NS-soil-specificity. **b** Dot plots represent S-root- and S-fruitbody-specific phylotypes, with dot color indicating mean relative abundance across different niches. Bars on the right indicate LDA (linear discriminant analysis) value with color indicating niche specificity (magenta = root, purple = fruitbody). Only bacterial phylotype with LDA ≥ 4 and fungal phylotype with LDA > 3.5 are displayed

To identify S-soil core phylotypes specific to S-soil in comparison with NS-soil, multiple analytical methods were employed. A phylotype was considered S-soil-specific if it was statistically significant in any analysis (LEfSe, ISA, and co-occurrence analysis) and consistently significant in at least two studies. Among S-core phylotypes, seven bacterial and four fungal phylotypes were S-soil-specific (Fig. 4a, Table 1, Table S7). Microbial phylotypes consistently showed S-soil specificity across geographic regions and host plants; some bacterial phylotypes exhibited significant S-soil specificity in both ST20 (Southwest China) and ST22 (Northeast China), while some fungal phylotypes showed S-soil-specificity in both oak-associated shiro (ST20) and pine-associated shiro (ST21). Even when statistical testing was not feasible, *Paraburkholderia* pt 108.3 (bacteria) and *Mucor zonatus* (fungi) consistently showed a higher abundance in S-soil compared to NS-soil. Although *U. dimorpha* showed low abundance in the S-soil of ST17 due to the dominance of TM, its consistent positive correlation with TM led to its identification as S-soil-specific. *Phellodon melaleucus* was identified as S-soil-specific but, at the same time, identified as NS-soil-specific in ST17.

**Table 1:** Microbial phylotypes associated with different niches of shiro. Close relatives list species belonging to the same phylotype. An asterisk at the end of a species name indicates that the phylogenetic tree was manually inspected to assign close relatives; otherwise, close relatives were assigned automatically. A hyphen indicates either that listing close relatives was unnecessary because the phylotype corresponds exactly to a named species, or that no confident close relatives could be assigned. Shiro core shows whether the phylotype is one of the shiro core phylotypes. Cultured denotes whether phylotypes have been detected via isolation in any studies on shiro microbes

**Niche specificity**	**Phylotype**	**Close relatives**	**Shiro core**	**Cultured**
S-soil-specific bacteria	*Roseiarcus* pt 148.8	*-*	Yes	No
	*Paraburkholderia*pt 108.3	*Par. rhynchosiae*, *Par. sartisoli*, *Par. megapolitana*, *Par. phytofirmans*, *Par. dipogonis*, *Par. pallidirosea*, *Par. sediminicola*	Yes	Yes
	*Paraburkholderia*pt 109.39	-	Yes	Yes
	*Caballeronia* pt 110.9	-	Yes	Yes
	*Caballeronia* pt 110.10.2	*Cab.* *udeis*	Yes	Yes
	*Acidipila* KB906735_s	-	Yes	No
	PAC000121_g pt 77.32	-	Yes	No
S-root-specific bacteria	*Acinetobacter* pt 100.30	*A.* *johnsonii*, *A.* *oryzae*	No	No
	*Ralstonia insidiosa*	-	No	No
	*Prosthecomicrobium*_g1AY138237_s	-	No	Yes
	*Sphingomonas oligophenolica*	-	Yes	No
	*Bradyrhizobium* pt 147.45	*Br.* *lupini*, *Br.* *yuanmingense*, *Br.* *cytisi*, *Br.* *vignae*	No	No
S-fruitbody-specific bacteria	*Pseudomonas*pt 97.28	*Ps.* *fluorescens*, *Ps.* *mediterranea*, *Ps.* *palleroniana*, *Ps.* *thivervalensis*, *Ps.* *veronii*, *Ps.* *prosekii*, *Ps.* *lini*, *Ps.* *brassicacearum*, *Ps.* *paralactis*, *Ps.* *helleri*, *Ps.* *yamanorum*, *Ps.* *endophytica*	Yes	Yes
	*Cupriavidus pauculus*	-	No	No
	*Ewingella americana*	-	No	Yes
	*Pseudomonas*pt 101.24	*Ps.* *jessenii*, *Ps.* *granadensis*, *Ps.* *helmanticensis*	Yes	Yes
	*Stenotrophomonas* pt 121.62	*St. rhizophila*	No	No
	*Sphingomonas* pt 156.52	*Sp.* pituitosa*, *Sp.* azotifigens*, *Sp.* trueperi*	No	No
	*Pedobacter* pt 17.M.28	-	No	No
	*Chryseobacterium* pt 34.7	*Chr. rhizosphaerae*	No	No
S-soil-specific fungi	*Umbelopsis dimorpha*	-	Yes	Yes
	*Mucor zonatus*	-	Yes	Yes
	*Phellodon melaleucus*	-	Yes	No
	Ascomycota pt 61.27	*-*	Yes	No
S-root-specific fungi	*Phialocephala* pt 33.50	Ph. fortinii*	Yes	Yes
	Ascomycota pt 61.M.427	-	No	No
	*Basidiodendron* pt 100.M.6	-	No	No
	*Cenococcum geophilum*	-	Yes	No
	*Paravamsapriya* pt 64.M.6	-	No	No
	*Dictyochaeta stratosa*	-	No	No
	*Lachnum* pt 34.28	La. Virgineum*	No	Yes
	*Geminibasidium* pt 66.M.2	-	No	No
	*Oidiodendron chlamydosporicum*	-	Yes	Yes
S-fruitbody-specific fungi	*Thelephora ganbajun*	-	No	No
	*Epicoccum* pt 24.5	E. dendrobii*, *E.* pruni*, *E.* endophyticum*, *E.* poae*, *E.* layuense*	No	No

We identified a few highly niche-specific phylotypes within shiro by comparing S-soil, S-root, and S-fruitbody using LEfSe (Fig. 4b, Table 1, Table S8). In the S-root bacterial community, *Acinetobacter* pt 100.30 and *Ralstonia insidiosa* were the most abundant bacterial phylotypes, comprising 71.4% and 16.3% of the total abundance, respectively. In the S-fruitbody bacterial community, *Pseudomonas* pt 97.28 (18.7%), *Cupriavidus pauculus* (14.2%), and *Ewingella americana* (11.3%) dominated. In the fungal communities of S-root and S-fruitbody, TM was the most abundant phylotype. Nevertheless, *Phialocephala* pt 33.50 was dominant in the S-root fungal community (12.0%) but was rare in S-soil (<1%), indicating its S-root specificity. *Thelephora ganbajun* and *Epicoccum* pt 24.5 were identified as S-fruitbody-specific fungi, though low in abundance.

The S-fruitbody microbiome exhibited a modular network structure that reflects distinct niche specificity of the phylotypes, as revealed by inter-kingdom co-occurrence analysis (Fig. S5, Table S9). The Markov clustering identified four distinct clusters, revealing two characteristic groups: a TM-positive and a TM-negative group. These groups were positively or negatively correlated with TM, respectively, and showed a strong negative correlation with each other. The TM-positive group harbored S-fruitbody-specific phylotypes, including *E. americana* (*r* = 0.71 with TM), *Pseudomonas* pt 97.28 (*r* = 0.71), and *Epicoccum* pt 24.5 (*r* = 0.66). In contrast, the TM-negative group harbored both S-fruitbody-specific and S-root-specific phylotypes, with S-root-specific *Ra. insidiosa* (*r* = −0.47) and *Acinetobacter* pt 100.30 (*r* = −0.44) serving as major hubs (degree = 33 and 31, respectively). Other negatively associated phylotypes included *Acinetobacter gerneri* (*r* = −0.43), *A. junii* (*r* = −0.53), and *Cupriavidus pauculus* (formerly *Ra. paucula*; *r* = −0.44). Among fungi, *T. ganbajun*, though not directly correlated with TM, was clustered within the TM-negative group.

### Functional differentiation of shiro microbes across niches

**Fig. 5:**
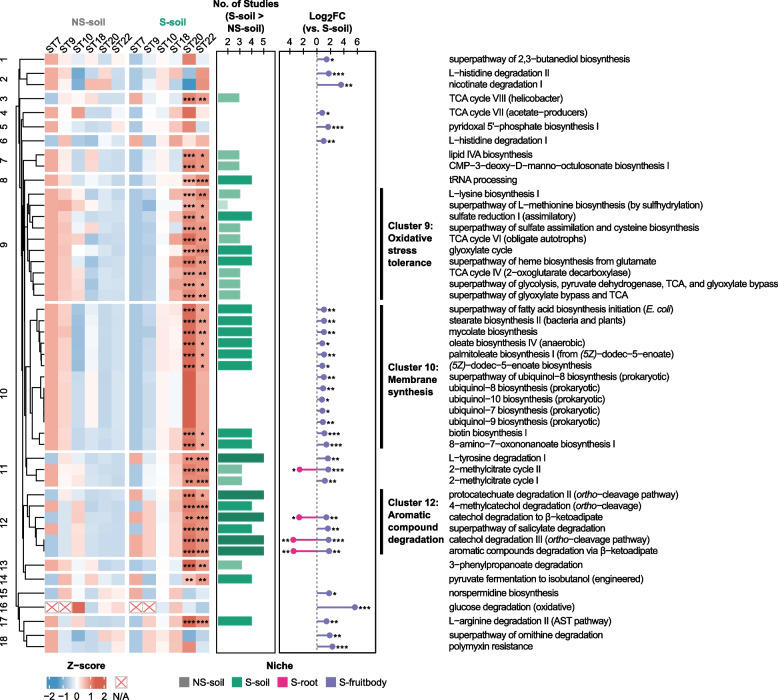
Predicted bacterial functions enriched in different niches of shiro. Heatmap shows z-scored expression levels of metabolic pathways (rows) across sample groups (columns), classified as NS-soil (grey) or S-soil (green), with red and blue indicating high and low expression, respectively. Study IDs are shown above the heatmap columns. Metabolic pathways are hierarchically clustered based on expression patterns, with cluster numbers shown on the far left. A histogram shows how consistently pathways are enriched in S-soil over NS-soil, with higher values indicating enrichment in a greater number of studies. A lollipop plot illustrates log2 fold-change of expression in S-root vs. S-soil (magenta) and S-fruitbody vs. S-soil (purple) comparisons, displaying only statistically significant values. Asterisks denote adjusted *p*-values of the DESeq2 results (*p ≤ 0.05, **p ≤ 0.01, ***p ≤ 0.001)

To identify functional differentiation in microbial communities across niches, we predicted and compared their functional profiles. Pairwise comparisons of S-soil with NS-soil, S-root, and S-fruitbody identified 49 enriched pathways (Fig. 5) and 417 enzymatic reactions (Table S10). Based on the predicted level of expression across samples, pathways were clustered into 18 clusters. Pathways related to oxidative stress tolerance (cluster 9)—including sulfate reduction, glyoxylate cycle, and heme biosynthesis—were specifically enriched in S-soil. Pathways related to membrane synthesis (cluster 10) were enriched in both S-soil and S-fruitbody. Pathways specifically enriched in S-fruitbody included superpathway of 2,3-butanediol biosynthesis, L-histidine degradation, nicotinate degradation I, TCA cycle VII (acetate-producers), pyridoxal 5’-phosphate biosynthesis I, norspermidine biosynthesis, glucose degradation (oxidative), superpathway of ornithine degradation, and polymyxin resistance.

**Fig. 6:**
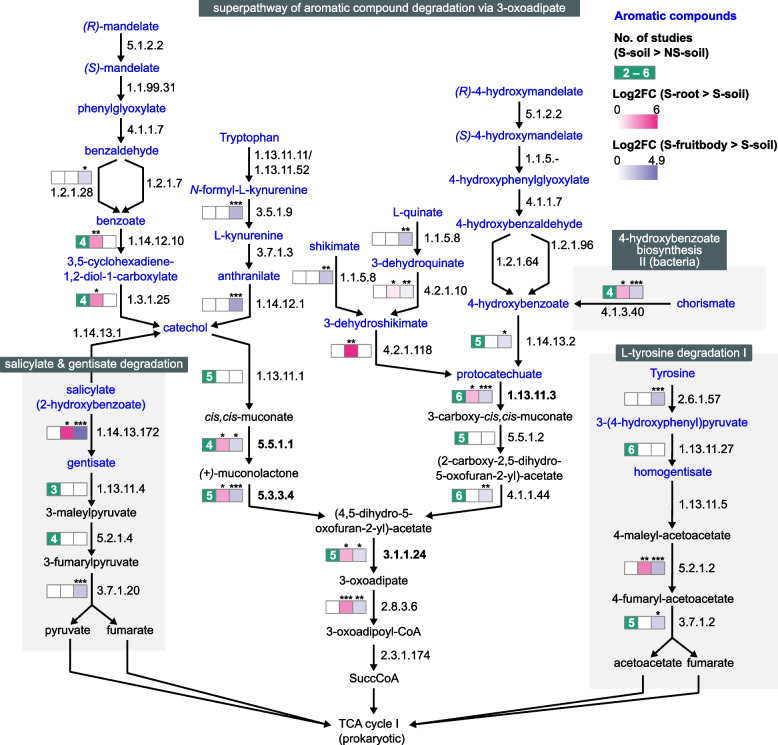
Aromatic compound degradation by bacterial communities across different niches of shiro. Compounds are labeled in black (non-aromatic) or blue (aromatic), and arrows indicate enzymatic reactions. Boxes on the left of the arrows summarize enrichment levels for each reaction: the green box shows how consistently a reaction is enriched in S-soil over NS-soil samples, with higher values indicating enrichment in a greater number of studies; the magenta and purple boxes represent log2 fold-changes of the expression in S-root vs. S-soil and S-fruitbody vs. S-soil comparisons, respectively. Asterisks above the boxes denote adjusted *p*-values of the DESeq2 results (*p ≤ 0.05, **p ≤ 0.01, ***p ≤ 0.001). EC numbers are represented on the right of the arrows, with EC numbers enriched in all niches highlighted in bold

Three aromatic compound degradation pathways (cluster 12) were enriched across all pairwise comparisons: catechol degradation to β−ketoadipate, catechol degradation III (ortho−cleavage pathway), and aromatic compounds degradation via β−ketoadipate. These three pathways were most consistently enriched in S-soil and showed the highest expression in the S-root. According to the hierarchy of MetaCyc pathways, these were classified under “superpathway of aromatic compound degradation via 3-oxoadipate (PWY-2504)” (Fig. 6). Key ring-cleavage reactions for protocatechuate (EC 1.13.11.3) and catechol (EC 1.13.11.1), along with downstream degradation to 3-oxoadipate, were consistently enriched in S-soil. Most of these reactions were further enriched in the S-root and S-fruitbody. By contrast, some reactions were enriched in specific niches: 3-dehydroshikimate degradation (EC 4.2.1.118) was exclusively enriched in the S-root (log2FC = 6.0), while salicylate-degrading reactions (EC 1.14.13.172) were enriched in both the S-root (log2FC = 5.6) and S-fruitbody (log2FC = 4.9).

For the fungal communities, five pathways were consistently enriched in S-soil compared to NS-soil across multiple studies, while two pathways were enriched in S-root compared to S-soil (Table S11). S-soil-enriched functions included mitochondrial biogenesis (ko03029), DNA replication proteins (ko03032), oxidative phosphorylation (ko00190), cardiac muscle contraction (ko04260), and cell adhesion molecules (ko04515). In S-root, antimicrobial resistance genes (ko01504) and bacterial motility proteins (ko02035) were enriched compared to S-soil.

### Validation of niche-specific bacterial functions

**Fig. 7:**
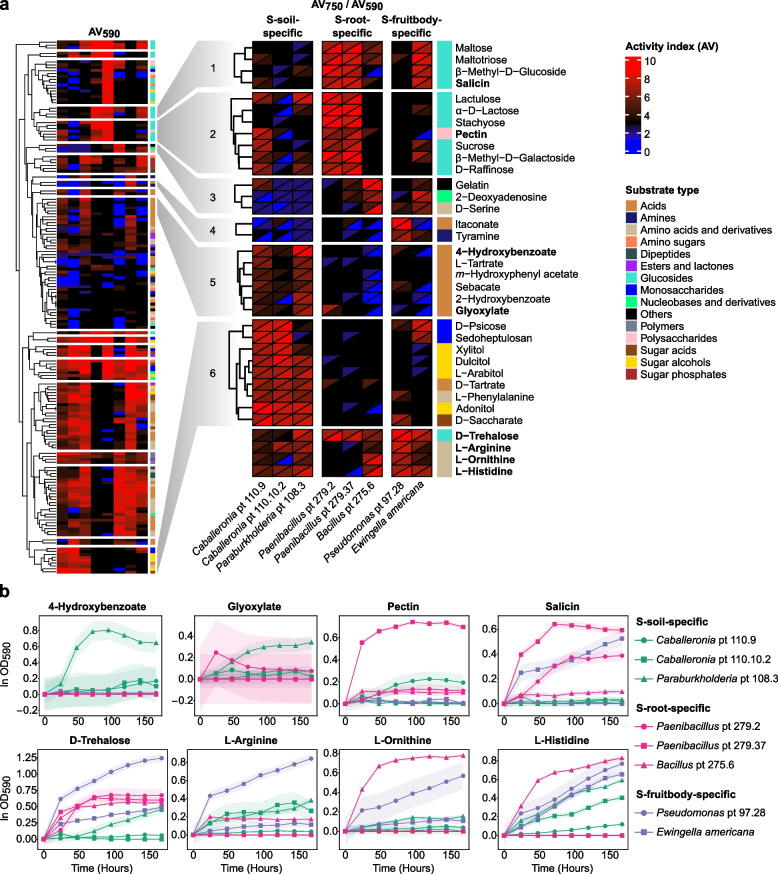
Substrate utilization ability of shiro bacteria. **a** Heatmap on the left depicts the activity index at 590 nm (AV590, cellular respiration) for each substrate (row) across bacterial phylotypes (column), with red and blue indicating high and low AV values, respectively. AV values of 3 (black) correspond to the activity in the negative control well (A1). Substrates were hierarchically clustered based on AV590. Substrate types are shown in different colors on the right of the triangular heatmap. The triangular heatmap displays both AV750 (cellular growth, up) and AV590 (down). Bacterial phylotypes are separated by their niche specificity. **b** Growth curves of bacterial phylotypes on selected substrates highlighted in bold on panel a. Absorbance at 590 nm was measured at 24-hour intervals. Each curve was normalized by subtracting the absorbance values of the negative control well (A1) for each strain

To examine whether individual phylotypes contribute to the predicted community-level functions, we tested the ability of eight niche-specific bacterial phylotypes to utilize 166 different substrates (Fig. 7). The tested strains included three representative strains from S-soil-specific core phylotypes, three from S-root-specific phylotypes (based on barcoding results), and two from S-fruitbody-specific phylotypes. S-soil-specific phylotypes primarily utilized substrates in cluster 5—six organic acids comprising three aromatic compounds (4-hydroxybenzoate, m-hydroxyphenyl acetate, and 2-hydroxybenzoate) and three non-aromatic compounds (L-tartrate, sebacate, and glyoxylate)—with *Paraburkholderia* pt 108.3 showing the strongest activity on these substrates, followed by *Caballeronia* strains. This utilization pattern coincided with the enrichment of aromatic compound degradation and glyoxylate cycle in S-soil (Fig. 5). In particular, *Paraburkholderia* pt 108.3 showed the best respiration and growth on 4−hydroxybenzoate compared to all other substrates and strains. S-soil-specific phylotypes also preferentially utilized substrates in cluster 6, including pentitols (xylitol, L-arabitol, and adonitol) and sedoheptulosan. The utilization of these substrates corresponds to the activation of the pentose phosphate pathway, supported by the consistent enrichment of transketolase activity in S-soil (Table S10).

S-root- and S-fruitbody-specific phylotypes showed distinct substrate preferences. S-root-specific *Paenibacillus* phylotypes primarily utilized plant-associated substrates such as salicin, stachyose, pectin, sucrose, and D-raffinose in clusters 1 and 2. These substrates were rarely utilized by other bacteria, with limited utilization observed in *Caballeronia* pt 110.9 and *E. americana*. While *Paenibacillus* strains efficiently utilized salicin, neither of them utilized 2-hydroxybenzoate (salicylate). S-fruitbody-specific *Pseudomonas* pt 97.28 efficiently utilized amino acids such as L-arginine, L-ornithine, and L-histidine, coinciding with the enrichment of amino acid degradation pathways in fruitbody (Fig. 5). *Pseudomonas* pt 97.28 exhibited the highest respiration and growth on itaconate and D-trehalose among all tested strains.

## Discussion

TM is widely distributed across Eurasia, and its associated microbes have been investigated from various geographic regions and niches. However, knowledge on shiro microbes has been fragmented; to address this, we integrated heterogeneous microbial sequences by applying phylogenetic placement-based phylotyping. Despite technical and geographic heterogeneity, we identified a set of shiro core microbial phylotypes that are essential for maintaining stable community function. In addition, we revealed niche-specific microbial phylotypes and functions across S-soil, S-roots, and S-fruitbodies, demonstrating clear niche differentiation. To our knowledge, no previous study has systematically compared shiro microbes within a unified phylogenetic and functional framework across the globe.

### Methodological biases reveal limited cultivation of shiro microbes

Comparison of metabarcoding and barcoding methods revealed overlaps and biases in detecting shiro microbes. Both methods frequently detected fast-growing r-selected bacteria from S-soil (*Caballeronia* and *Paraburkholderia*) and fruitbodies (*Ewingella* and *Pseudomonas*). Their rapid growth and ease of cultivation are likely supported by enriched membrane synthesis, consistent with the regulatory role of membrane lipid biosynthesis in bacterial growth [82]. However, discrepancies between the methods were also evident. Barcoding overrepresented Gram-positive bacteria capable of forming resistant structures, such as endospores in *Bacillus* and *Paenibacillus*, or desiccation-tolerant spores in *Streptomyces*. This pattern likely reflects their resistance to surface sterilization rather than their true abundance [83]. Supporting this, fluorescence in situ hybridization (FISH) analyses showed low abundance of Bacilli in both EcM tissue [84] and ectomycorrhizosphere [85]. These methodological biases emphasize the need for targeted isolation of uncultured core and niche-specific phylotypes to better synthesize the natural shiro microbiome—an essential step toward its artificial cultivation.

### Spatiotemporal persistence of shiro microbiome

We identified shiro core microbial phylotypes conserved across the globe. Although few in number, shiro core phylotypes accounted for a considerable proportion of the total community abundance. Given taxonomic revisions over time, some of the phylotypes (*Caballeronia*, *Paraburkholderia*, and Mucoromycota) may have been misclassified in earlier studies. For example, the members of *Pseudomonas* frequently isolated by H. Ohara and M. Hamada [14] in front of the shiro may correspond to *Caballeronia* or *Paraburkholderia*, which were previously classified as *Pseudomonas* [86]. Similarly, TM has often been associated with *Mortierella* sp. [14, 21, 30, 37] and *Mortierella vinacea* (= *U. vinacea*) [29, 87], but we re-identified many of these *Mortierella* sequences as *Umbelopsis dimorpha*, indicating historical misclassification [88–90]. The spatiotemporal consistency of shiro core phylotypes reinforces the idea that these microbes contribute to key ecological processes essential for TM survival and growth.

### Niche differentiation of microbes in shiro soil, root, and fruitbodies

We identified geographically consistent S-soil-specific phylotypes potentially involved in TM growth, as previously tested in studies assessing their promotive or inhibitory effects [22, 23, 29, 31]. *Caballeronia* and *Paraburkholderia* species inhibited TM hyphal growth in vitro [22, 31], yet their role as mycorrhizal helper bacteria (MHB) cannot be ruled out. For instance, *Paraburkholderia fungorum* showed inhibitory or neutral effects on EcM fungi when supplied with monosaccharides, polysaccharides, and sugar alcohols, but promoted mycelial growth when supplemented with organic acids as a sole carbon source [91]. Indeed, *Paraburkholderia* pt 108.3 efficiently utilized certain organic acids. This suggests that previous attempts to test MHB effects on media with glucose as a primary carbon source could be biased [22, 31]. Using alternative media will allow for a more accurate assessment of their potential as MHBs for TM. On the other hand, mycelial extracts of *U. dimorpha* and *Mu. zonatus* promoted TM growth in vitro [23], and *U. vinacea* (= prob. U. dimorpha) showed no inhibition in filtrate or co-culture assays [29], suggesting their potential as mycorrhiza helper fungi (MHF). Another intriguing fact is that *Umbelopsis* and *Mucor* can endosymbiotically harbor *Paraburkholderia*; by sheltering this plant growth-promoting rhizobacterium within its hyphae, S-soil-specific fungi may enhance plant health [92]. Therefore, it would be interesting to figure out how they interact with TM as symbionts.

Following Proteobacteria (e.g., *Caballeronia* and *Paraburkholderia* phylotypes), our comprehensive analysis revealed that Acidobacteria comprise a core component of the S-soil bacterial community. Bacteria were once thought to be absent in active mycorrhizal zones (AMZ) of S-soil, where fruitbodies occur, due to antimicrobial compounds [14, 21]. However, culture-independent barcoding approaches (e.g., PCR-DGGE) revealed the presence of Acidobacteria in AMZ [21], which can comprise up to 84.4% of the total bacterial abundance in AMZ [28]. Among Acidobacteria, *Acidipila* KB906735_s and *Edaphobacter aggregans* stood out as S-soil core phylotypes, with the former being S-soil-specific. Both taxa belong to Acidobacteria subdivision 1, a group characterized by the highest lignocellulolytic activity in acidic soil [93, 94]. Indeed, *Acidipila* KB906735_s can degrade recalcitrant plant polymers [95], and *Acidipila* plays a key role in coniferous wood decomposition [96]. Their persistence in S-soil may be attributed to their ability to utilize recalcitrant plant materials abundant in S-soil and senescent S-roots, such as lignin, hemicellulose, and tannins [20, 97]. In addition, the occasional dominance of Acidobacteria in AMZ is possibly due to their adaptations to the acidic conditions caused by oxalate accumulation [19]. Their dominance in AMZ may be further supported by their resistance to antimicrobial compounds, potentially mediated by CAZymes. Altogether, Acidobacteria may play a potential role in nutrient cycling through the decomposition of recalcitrant plant materials, with their tolerance to acidity and antimicrobial compounds likely providing a competitive advantage in AMZ.

S-root and S-fruitbody were dominated by a few highly niche-specific phylotypes, and co-occurrence analysis further supported their niche specificity. *Acinetobacter* pt 100.30 and *Ra. insidiosa* dominated the S-root and showed a strong positive correlation with each other, suggesting they may function as an S-root-specific module. Moreover, these taxa and their close relatives appear to be generally associated with mycorrhiza. For example, *Acinetobacter proteolyticus* was found to dominate S-root [98], *Acinetobacter* cf. *johnsonii* was detected in other EcM root tips [99], and *A. johnsonii *and *Ra. insidiosa* were isolated from arbuscular mycorrhizal spores [100]. Their consistent association with mycorrhiza suggests their potential role in mycorrhizal development.

The roles of S-root-specific fungi are not fully understood, yet there is a possible explanation. M. Ogawa [18] frequently isolated non-spore-forming root fungi—possibly including *Ph. fortinii* and *L. virgineum*—from decaying S-root tips rather than from fresh ones. These fungi exhibit cellulolytic and lignolytic activities, implying a functional role in degrading senescent roots [18, 101]. This aligns with previous reports of a negative correlation between *Ph. fortinii* and TM [20]. Microscopic analysis further confirmed the presence of root-endophytic fungi in S-root tips, some forming microsclerotia likely produced by *Ph. fortinii* [101, 102]. Collectively, S-root-specific fungi, particularly *Ph. fortinii*, are likely to play a key role in degrading senescent S-roots.

S-fruitbody-specific phylotypes included both potentially beneficial and detrimental microbes for TM. Among bacteria, *E. americana*, *Pseudomonas* pt 97.28, and *Pseudomonas* 101.24 exhibited positive correlations with TM within the S-fruitbody, suggesting they may synchronously grow with TM mycelia. The promotion of TM mycelial growth [40, 103] and inhibition of S-fruitbody-inhabiting molds [40] support their potential roles as mycorrhizal helper bacteria (MHB). *Ewingella americana* generally causes brown blotch on cultivated mushrooms [104], but it can also be nonpathogenic, even antagonizing mycopathogenic bacteria [105]. Among fungi, *Epicoccum* pt 24.5 was positively correlated with TM. Given that closely related *Epicoccum* spp. are non-pathogenic plant endophytes with antifungal potential [106–108], *Epicoccum* pt 24.5 may serve as a biocontrol agent against S-fruitbody pathogens. In contrast, *Thelephora ganbajun* was clustered within the TM-negative group and may compete with TM. This species, endemic to Yunnan Province, forms ectomycorrhizal associations with *Pinus yunnanensis* and *Pinus kesiya* [109]. *Thelephora* was also the most abundant taxon in the inner tissue of S-fruitbody, supporting its potential role as a strong competitor of TM [39]. Together with *Phellodon melaleucus*, these EcM fungi belonging to Thelephorales may compete with TM for the same host plant [109]. Supporting this, *Phellodon niger* was reported to be aggressively harmful to TM [110].

### Distinct functions of shiro microbes across different niches

Functional prediction of bacterial communities, together with substrate utilization tests of individual bacterial phylotypes, consistently showed enrichment of aromatic compound degradation in S-soil. Aromatic compound degradation was predicted to gradually increase from NS-soil to S-soil to S-roots, suggesting that S-roots could be the primary source of the aromatic compounds. Indeed, plant root tips rapidly accumulate diverse aromatic compounds (e.g., catechin, epicatechin, 4-hydroxybenzoate glucoside, 4-hydroxybenzaldehyde, and vanillin) during the early stage of EcM colonization to prevent fungal penetration [111, 112]. These aromatic compounds can be degraded by bacteria via catechol and protocatechuate, eventually entering the TCA cycle as 3-oxoadipate [113, 114]. The ability of S-soil-specific core phylotypes *Caballeronia* 110.9 and *Paraburkholderia* pt 108.3 to utilize 4-hydroxybenzoate suggests that they can also degrade other EcM root tip-derived aromatic compounds through protocatechuate. This aligns with previous reports of these genera being principal degraders of 4-hydroxybenzoate in forest soils [115]. Therefore, S-soil and S-root-specific bacteria likely degrade defensive aromatic compounds from the host plant, potentially promoting the colonization of TM in its early stages. Indeed, certain bacteria can promote EcM fungal growth by degrading aromatic compounds that inhibit the fungal growth [116]. Aromatic compounds released from S-roots may further stimulate *Paraburkholderia* to decompose soil organic matter, thereby supplying nutrients to both TM and its host plant [115].

Pathways involved in oxidative stress tolerance were specifically enriched in the S-soil bacterial communities, reflecting adaptation to oxidative stress generated during aerobic degradation of aromatic compounds [117]. Supporting this, bacteria capable of degrading aromatic compounds often possess defense mechanisms like glutathione biosynthesis and the glyoxylate cycle, which help mitigate oxidative stress [117, 118]. Moreover, S-soil bacteria showed enrichment of transketolase and the utilization of five- and seven-carbon compounds, indicating an enhanced pentose phosphate pathway which provides abundant reducing power (NADPH) to cope with oxidative stress. Fungal communities of S-soil also exhibited enrichment of oxidative phosphorylation and mitochondrial biogenesis, supporting the adaptation of S-soil-inhabiting microbes to high oxidative stress. This adaptation also coincides with the fact that TM thrives in well-aerated sandy soils [3].

The dominance of a few S-root-specific bacterial phylotypes likely reflects the strong selectivity exerted by plant-derived compounds, such as salicylate. These compounds affect root-associated bacterial communities by serving as growth signals or carbon sources [119, 120]. Increased salicylate-5-hydroxylase (S5H) activity in S-root bacteria suggests their ability to tolerate host defense responses. *Ralstonia* species are known for their S5H activity [121–123], while *A. johnsonii* can also utilize salicylate [124], though the presence of S5H is uncertain in *Acinetobacter* [125, 126]. By contrast, *Paenibacillus* phylotypes, frequently isolated from the S-roots, utilized various plant-derived substrates, including salicin, pectin, and oligosaccharides, but not salicylate. This suggests metabolic niche differentiation between salicylate-utilizing and salicin-utilizing root-associated bacteria [127]. The role of *Paenibacillus* in EcM colonization remains unclear [128], but we propose that *Paenibacillus* may aid Hartig net formation by degrading apoplastic pectin, since pectinase is essential for EcM fungi to penetrate a host plant [129]. Although rarely detected through metabarcoding, *Paenibacillus* is an important MHB [128] that has either promoted or shown a neutral effect on the mycelial growth of TM in vitro—never an inhibitory one [22, 31, 40].

S-fruitbody bacterial community was specifically enriched with amino acid catabolic pathways, and S-fruitbody-specific *Pseudomonas* pt 97.28 rapidly utilized amino acids such as L-arginine, L-ornithine, and L-histidine, as well as D-trehalose. The ability to metabolize trehalose is crucial in the context of EcM interactions since trehalose accumulation in EcM fungal hyphae can attract P. fluorescens, which, in turn, produces thiamine that promotes fungal growth in vitro [130]. Supporting this, trehalose-utilizing *P. fluorescens* strains promoted or mildly inhibited EcM fungal growth, whereas non-utilizing strains exerted strong inhibitory effects [131]. Taken together, the trehalose-utilizing ability of *Pseudomonas* pt 97.28 may be crucial for its function as an MHB for TM. Ironically, *Pseudomonas* pt 97.28 may contribute to the degradation of the fruitbody. As fruitbodies mature and detach from the mycelium, L-arginine is metabolized into L-ornithine and urea to meet the nutritional demands of fruitbody development [132, 133]. Thus, *Pseudomonas* pt 97.28 likely thrives in fruitbodies by utilizing these compounds. Similarly, arginine and ornithine degradation pathways, as well as *Pseudomonas*, were reported to increase in rotting mushrooms [134]. In addition, biosynthesis of 2,3-butanediol, a compound known to accumulate as mushrooms ferment or decay [135, 136], was predicted to be enriched in the S-fruitbody. Collectively, S-fruitbody-specific bacteria are likely beneficial during early fruitbody development but may accelerate fruitbody decay as it matures.

### Limitations

Due to the inherent rarity of TM, some studies lacked sufficient samples for robust statistical analyses. Nevertheless, shiro core phylotypes and enriched functions exhibited consistent patterns across studies. As part of our validation efforts, one representative strain was selected for each phylotype. This approach may not fully capture the functional diversity within each phylotype, and we couldn’t validate the predicted functions involving uncultured microbes. In the case of fungi, the functional roles of TM may have been overrepresented within the S-soil. Despite these limitations in functional investigation, the selected bacterial strains adequately represented several key microbiome functions.

## Conclusion

This study systematically integrated all available shiro microbial sequences to identify core and niche-specific microbes. Despite technical and geographic variation, stable shiro core microbial phylotypes were identified, along with niche-specific phylotypes and metabolic functions. These findings provide a unified ecological framework for understanding TM–microbe interactions and support future research and cultivation efforts. Future research may focus on isolating key microbial phylotypes, uncovering their ecological roles in symbiosis and fruiting, and optimizing microbial interactions for TM cultivation. These efforts will not only advance our understanding of shiro microbes but also extend ecological insights into the microbial ecology of other EcM fungi.

## Supplementary Information

Supplementary Material 1: Supplementary Table S1. Sampling locations and the number of shiro samples collected in this study. Supplementary Table S2. Reanalyzed studies on shiro microbes. Supplementary Table S3. Summary of key studies investigating shiro microbes without publicly available sequence data. Supplementary Table S4. Taxonomic assignment of shiro bacterial sequences. Supplementary Table S5. Taxonomic assignment of shiro fungal sequences. Supplementary Table S6. Shiro core microbial phylotypes. Supplementary Data S7. Niche-specific taxa distinguishing S-soil and NS-soil. Analytical indices include LEfSe scores, ISA values, and co-occurrence index for each study. Supplementary Table S8. S-root- and S-fruitbody- specific taxa detected using LEfSe among S-soil, S-root, and S-fruitbody samples. Supplementary Table S9. Phylotypes representing significant correlation with *Tricholoma matsutake* (|r| ≥ 0.35). Supplementary Table S10. Predicted enzymatic activities enriched in bacterial communities of different niches. Supplementary Table S11. Predicted metabolic pathways (KEGG) enriched in fungal communities of different niches. Overlapped functions among studies are highlighted in bold.

Supplementary Material 2.

Supplementary Material 3: Supplementary Figure S1. Workflow for microbial sequence analysis. Metabarcoding and barcoding sequences were preprocessed, filtered, and trimmed into query sequences. Database sequences (16S for bacteria and ITS for fungi) were aligned, and phylogenetic trees were inferred from the sequence alignments. The query sequences (blue lines) were placed into the backbone alignment and backbone tree. The query placed tree was decomposed and refined for accurate clustering and taxonomic assignment of the query sequences. For each refined subtree, sequences were clustered based on the branch length. Taxonomy was assigned either as a species name or as an LCA-derived phylotype, represented as pt x.y or pt x.y.z, with x,y, and z indicating a subtree, cluster, and subcluster. For example, cluster 1 was directly assigned to species A, while cluster 2 was assigned as a phylotype of LCA(C,D). Supplementary Figure S2. Read count distribution across metabarcoding studies. Bacterial and fungal samples are shown in (a) and (b), respectively. Samples were grouped by study and niche. Colors indicate different niches. Dots represent read counts of individual samples. Read count is displayed on log10 scale. The numbers above the box plots indicate the number of samples for each group. Supplementary Figure S3. Alpha diversity comparison of microbial communities between S-soil and NS-soil samples. Results for bacteria and fungi are shown in (a) and (b), respectively. Each panel represents a separate metabarcoding study. Differences in Shannon index between niches were assessed using the Wilcoxon rank-sum test, with asterisks indicating statistical significance (*p ≤ 0.05, **p ≤ 0.01, ***p ≤ 0.001). Supplementary Figure S4. Beta diversity comparison of microbial communities between S-soil and NS-soil samples. Bacterial and fungal communities are shown in (a) and (b), respectively. Each panel represents a separate metabarcoding study, with points indicating individual samples colored by niche. Samples were reclassified as S-soil or NS-soil based on TM read abundance using univariate clustering. Weighted UniFrac dissimilarity was used to determine distances between samples, and differences between niches were assessed using PERMANOVA (R2 and adjusted *p*-values). Supplementary Figure S5. Inter-kingdom co-occurrence analysis of microbial phylotypes in fruitbody samples. Dotted lines define clusters identified using the Markov clustering algorithm (MCL). Names of phylotypes are colored by niche specificity. Node stroke is colored by correlation with TM (green: TM-positive, brown: TM-negative). Node size corresponds to the number of connected nodes (degree). Edges indicate significant correlations between phylotypes (|r| ≥ 0.35), with thickness and color intensity being proportional to the absolute correlation strength.

## Data Availability

Metabarcoding data generated in this study have been deposited in the NCBI Sequence Read Archive under BioProject PRJNA1266365 (bacteria) and PRJNA1266404 (fungi). Barcoding data generated in this study have been deposited in the NCBI GenBank under accession numbers PV678996-PV679701 (bacteria) and PV688728-PV689520 (fungi).
