# Supplementary material for: Functionally distinct core microbes of Tricholoma matsutake revealed by cross-study analysis
Source: Microbiome. 2026 Feb 4;14:58. doi: 10.1186/s40168-025-02329-x (PMC12874918; doi:10.1186/s40168-025-02329-x)
Supplement: Supplementary file 2 — Supplementary Material 2. [file 40168_2025_2329_MOESM2_ESM.docx]

**Cross-study discovery of functionally distinct core microbes of *Tricholoma matsutake***

Shinnam Yoo^1^, Chang Wan Seo^1^ & Young Woon Lim^1, *^

^1^ School of Biological Sciences and Institute of Biodiversity, Seoul National University, Seoul, 08826, Republic of Korea

*Corresponding author: Young Woon Lim, E-mail: ywlim@snu.ac.kr

**Supplementary Methods**

***Sampling and sequencing of microbes***

A total of 20 shiro samples were collected across seven regions of South Korea during September 2020 and April to May 2021 **(Table S1)**. The distance between individual shiro samples in each region was at least 15 m. After removing the organic layer, S-soil and S-roots were collected in a size of 15 × 15 × 15 cm using a knife. Samples were individually stored in plastic bags at 4 °C and maintained at this temperature for up to 48 hours until processing. S-roots were separated from S-soil using a 2 mm sieve and S-root tips were morphologically identified. To verify the presence of TM in shiro samples, DNA was individually extracted from at least five fresh S-root tips (type Ⅰ described by WM Gill, A Guerin-Laguette, F Lapeyrie and K Suzuki [1]) for each sample using Instagene™ Matrix (Bio-Rad, Hercules, CA, USA). PCR was conducted using TM-specific primers (DTmF, DTmR) under the conditions described in JH Kim and YH Han [2], followed by visual inspection of the PCR product on 1% agarose gel (BIOFACT, Daejeon, the Republic of Korea) stained with the EcoDye DNA staining solution (SolGent Co., Daejeon, the Republic of Korea).

Bacterial and fungal communities were investigated using both metabarcoding and barcoding approaches. For the metabarcoding approach, bacterial communities of S-soil and S-root tips were investigated using PacBio Sequel, while fungal communities of S-roots were investigated using Illumina MiSeq. DNeasy® PowerSoil® Kit (Qiagen, Netherlands) was used for DNA extraction, following the manufacturer’s guidelines. Approximately 200 mg of S-soil, 50 mg of S-roots, and 10 fresh S-root tips were utilized for each sample, respectively. S-root tips were directly put into the bead containing tubes, while S-roots were ground in liquid nitrogen using mortar and pestle, followed by bead beating. 16S rDNA and ITS2 region were amplified with 27F/1492R and 5.8S-Fun/ITS4-Fun [3] primer sets for bacterial and fungal metabarcoding, respectively. For multiplexing, distinct adaptor sequences were added to the end of the primer. PCR was proceeded in two steps: The first round was proceeded 95 °C for 5 min, followed by 25 cycles at 95 °C for 40 s, at 55 °C for 40 s, and at 72 °C for 1 min, with a final extension at 72 °C for 5 min. In the second round, distinct adaptor sequences were attached to the primers for multiplexing. This round followed the condition of the first round, except with a reduced cycle number of 15. For the S-root samples, 5 cycles were added to the second round. PCR products were purified using an ExpinTM PCR SV kit (GeneAll Biotechnology, Seoul, South Korea).

For barcoding approach, bacteria were isolated using TSA and R2A, while fungi were isolated using PDA and DRBC media (Difco, Detroit, MI, USA). Each sample was separated into multiple niches: S-bulk soil, S-rhizosphere soil, and S-roots (S-root tips for bacteria). For the S-bulk soil, 5 g of sieved soil was mixed with 50 mL of sterilized water, diluted at a ratio of 1/100, and then 100 µL of the diluted solution was spread onto the media. S-rhizosphere soil was prepared by removing soil clumps from roots, submerging the roots in sterilized water in a 50 mL tube, vortexing for 1 min, and sonicating for 1 min using a CPX3800H-E sonicator (Branson, USA). Subsequently, 100 µL of the diluted suspension was spread on each media. S-roots and S-root tips were surface sterilized individually by immersion in 3 % H_2_O_2_ for 1 min, followed by three washes with sterilized water. After sterilization, eight pieces of samples (≤ 1 cm) were inoculated at regular distances onto three media for each type of medium. After 3 to 7 days of incubation at 25 ℃, bacteria and fungi were transferred to TSA and PDA, respectively, to obtain pure cultures. DNA was extracted from the isolates using the AccuPrep Genomic DNA Extraction Kit (Bioneer, Daejeon, Korea). To amplify 16S rDNA region (V1V4) of bacteria and ITS region of fungi, 27F/1492R [4] and ITS1F/ITS4 [5] primer sets were used, respectively. PCR was proceeded with initial denaturation at 95 °C for 5 min, followed by 35 cycles of denaturation at 95 °C for 40 s, annealing at 55 °C for 40 s, and extension at 72 °C for 1 min, with a final extension at 72 °C for 5 min. The PCR product was purified using the ExoSAP-IT™ (ThermoFisher, USA), and Sanger sequencing was performed by Macrogen (Seoul, Korea).

***Modifications for phylogenetic refinement***

After decomposing the query-placed tree into chunks with 500 tips each, refinement was performed using uDance [6] with the following modifications: (1) only “decomposition”, “gene tree inference”, refine”, and “stitch” steps were executed, (2) before gene tree inference, sequences were aligned using MAFFT v7.525 with --auto option (--globalpair for fungi) [7] and trimmed using trimAl v1.4.rev15 with -gt 0.02 and -cons 60 options [8], (3) long-branches were not removed during “gene tree inference” to avoid discarding valid query sequences that may appear as long branches. Following these modifications, the query-placed tree was decomposed into chunks of approximately 500 tips, and resulting subtrees were refined for each chunk using RAxML-NG with three starting trees and the GTR + G model [9].

**References**

1. Gill WM, Guerin-Laguette A, Lapeyrie F, Suzuki K. Matsutake – morphological evidence of ectomycorrhiza formation between *Tricholoma matsutake* and host roots in a pure *Pinus densiflora* forest stand. New Phytologist. 2000;147(2):381-8.

2. Kim JH, Han YH. Development of Specific Primer for *Tricholoma matsutake*. Mycobiology. 2009;37(4):317-9.

3. Taylor DL, Walters WA, Lennon NJ, Bochicchio J, Krohn A, Caporaso JG, et al. Accurate estimation of fungal diversity and abundance through improved lineage-specific primers optimized for Illumina amplicon sequencing. Applied and Environmental Microbiology. 2016;82(24):7217-26.

4. Weisburg WG, Barns SM, Pelletier DA, Lane DJ. 16S ribosomal DNA amplification for phylogenetic study. Journal of bacteriology. 1991;173(2):697-703.

5. White TJ, Bruns T, Lee S, Taylor J. Amplification and direct sequencing of fungal ribosomal RNA genes for phylogenetics. PCR protocols: a guide to methods and applications. 1990;18(1):315-22.

6. Balaban M, Jiang Y, Zhu Q, McDonald D, Knight R, Mirarab S. Generation of accurate, expandable phylogenomic trees with uDance. Nature Biotechnology. 2023.

7. Katoh K, Standley DM. MAFFT multiple sequence alignment software version 7: improvements in performance and usability. Molecular biology evolution. 2013;30(4):772-80.

8. Capella-Gutiérrez S, Silla-Martínez JM, Gabaldón T. trimAl: a tool for automated alignment trimming in large-scale phylogenetic analyses. Bioinformatics. 2009;25(15):1972-3.

9. Kozlov AM, Darriba D, Flouri T, Morel B, Stamatakis A. RAxML-NG: a fast, scalable and user-friendly tool for maximum likelihood phylogenetic inference. Bioinformatics. 2019;35(21):4453-5.
