# Supplementary material for: Functionally distinct core microbes of Tricholoma matsutake revealed by cross-study analysis
Source: Microbiome. 2026 Feb 4;14:58. doi: 10.1186/s40168-025-02329-x (PMC12874918; doi:10.1186/s40168-025-02329-x)
Supplement: Supplementary file 3 — Supplementary Material 3: Supplementary Figure S1. Workflow for microbial sequence analysis. Metabarcoding and barcoding sequences were preprocessed, filtered, and trimmed into query sequences. Database sequences (16S for bacteria and ITS for fungi) were aligned, and phylogenetic trees were inferred from the sequence alignments. The query sequences (blue lines) were placed into the backbone alignment and backbone tree. The query placed tree was decomposed and refined for accurate clustering and taxonomic assignment of the query sequences. For each refined subtree, sequences were clustered based on the branch length. Taxonomy was assigned either as a species name or as an LCA-derived phylotype, represented as pt x.y or pt x.y.z, with x,y, and z indicating a subtree, cluster, and subcluster. For example, cluster 1 was directly assigned to species A, while cluster 2 was assigned as a phylotype of LCA(C,D). Supplementary Figure S2. Read count distribution across metabarcoding studies. Bacterial and fungal samples are shown in (a) and (b), respectively. Samples were grouped by study and niche. Colors indicate different niches. Dots represent read counts of individual samples. Read count is displayed on log10 scale. The numbers above the box plots indicate the number of samples for each group. Supplementary Figure S3. Alpha diversity comparison of microbial communities between S-soil and NS-soil samples. Results for bacteria and fungi are shown in (a) and (b), respectively. Each panel represents a separate metabarcoding study. Differences in Shannon index between niches were assessed using the Wilcoxon rank-sum test, with asterisks indicating statistical significance (*p ≤ 0.05, **p ≤ 0.01, ***p ≤ 0.001). Supplementary Figure S4. Beta diversity comparison of microbial communities between S-soil and NS-soil samples. Bacterial and fungal communities are shown in (a) and (b), respectively. Each panel represents a separate metabarcoding study, with points indicating individua [file 40168_2025_2329_MOESM3_ESM.pptx]

## Slide 1
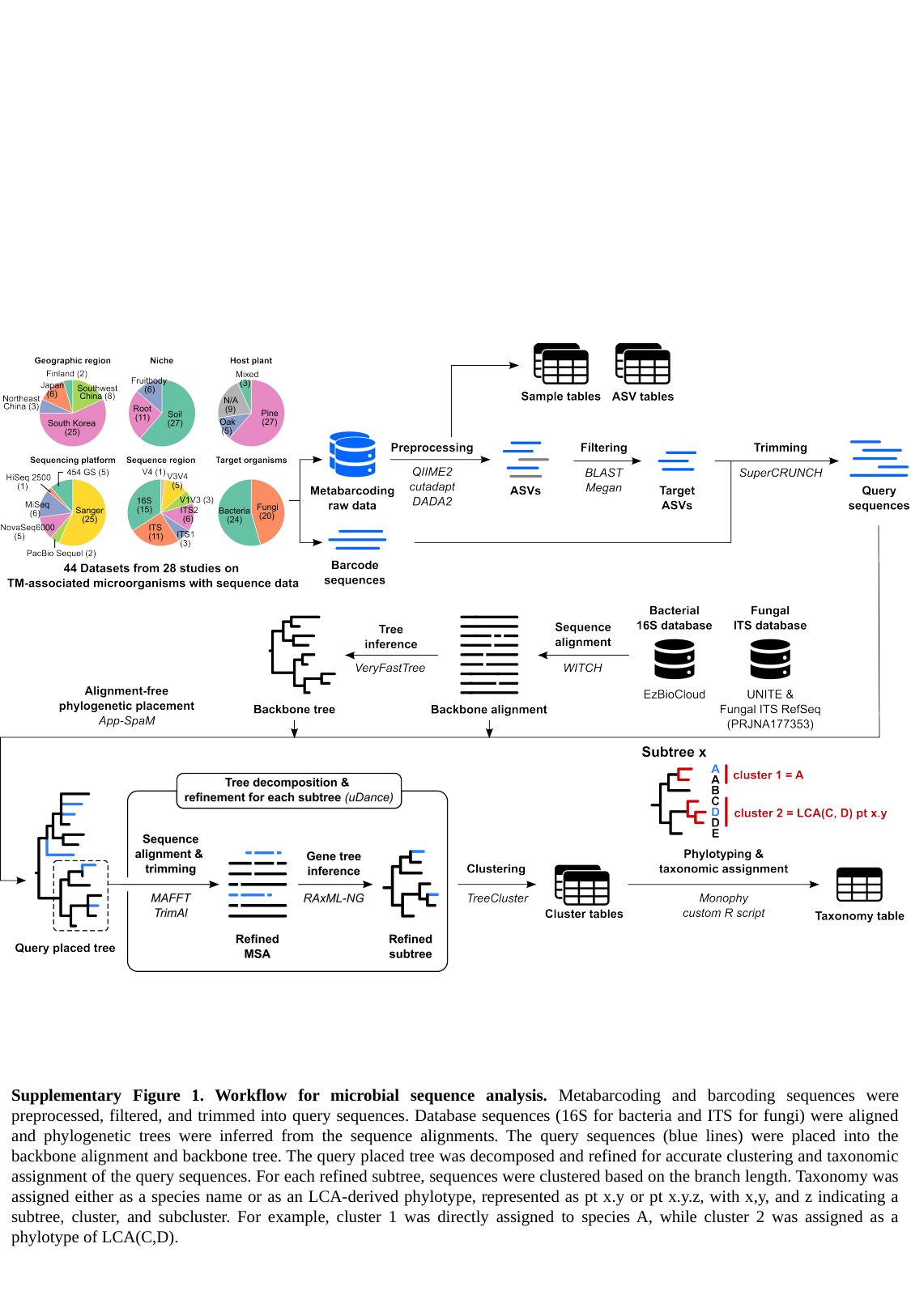

Supplementary Figure 1. Workflow for microbial sequence analysis. Metabarcoding and barcoding sequences were preprocessed, filtered, and trimmed into query sequences. Database sequences (16S for bacteria and ITS for fungi) were aligned and phylogenetic trees were inferred from the sequence alignments. The query sequences (blue lines) were placed into the backbone alignment and backbone tree. The query placed tree was decomposed and refined for accurate clustering and taxonomic assignment of the query sequences. For each refined subtree, sequences were clustered based on the branch length. Taxonomy was assigned either as a species name or as an LCA-derived phylotype, represented as pt x.y or pt x.y.z, with x,y, and z indicating a subtree, cluster, and subcluster. For example, cluster 1 was directly assigned to species A, while cluster 2 was assigned as a phylotype of LCA(C,D).

## Slide 2
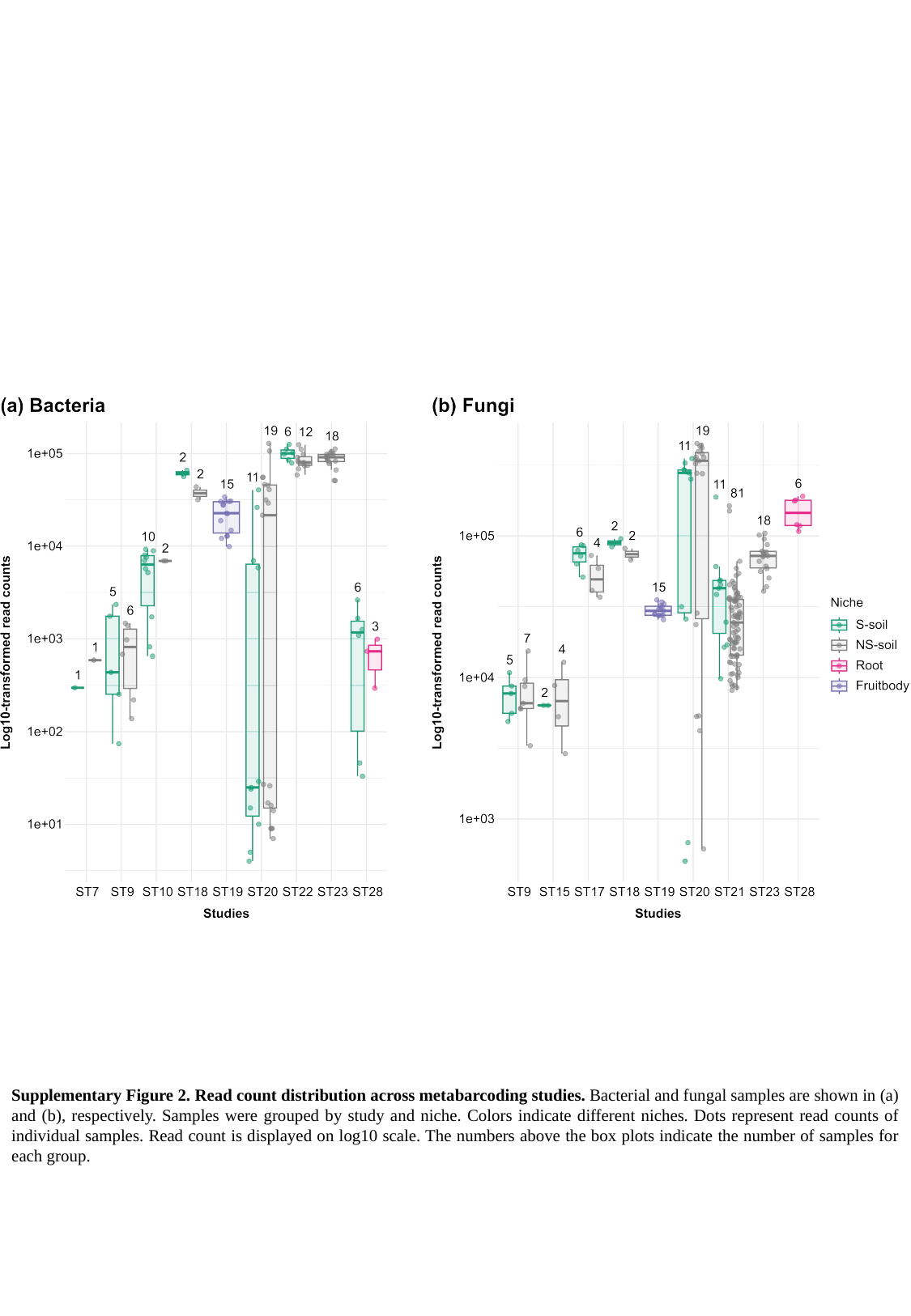

Supplementary Figure 2. Read count distribution across metabarcoding studies. Bacterial and fungal samples are shown in (a) and (b), respectively. Samples were grouped by study and niche. Colors indicate different niches. Dots represent read counts of individual samples. Read count is displayed on log10 scale. The numbers above the box plots indicate the number of samples for each group.

## Slide 3
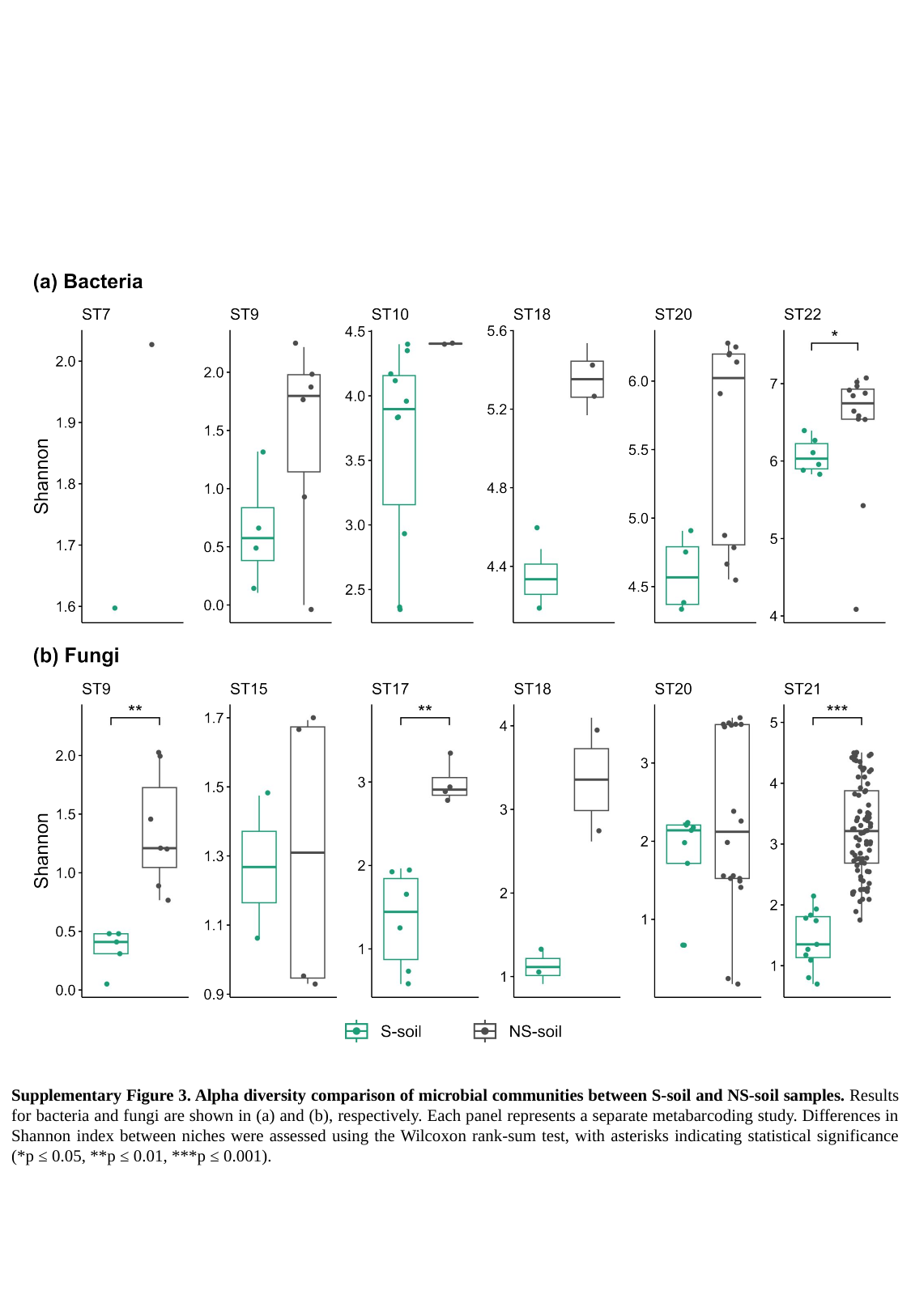

Supplementary Figure 3. Alpha diversity comparison of microbial communities between S-soil and NS-soil samples. Results for bacteria and fungi are shown in (a) and (b), respectively. Each panel represents a separate metabarcoding study. Differences in Shannon index between niches were assessed using the Wilcoxon rank-sum test, with asterisks indicating statistical significance (*p ≤ 0.05, **p ≤ 0.01, ***p ≤ 0.001).

## Slide 4
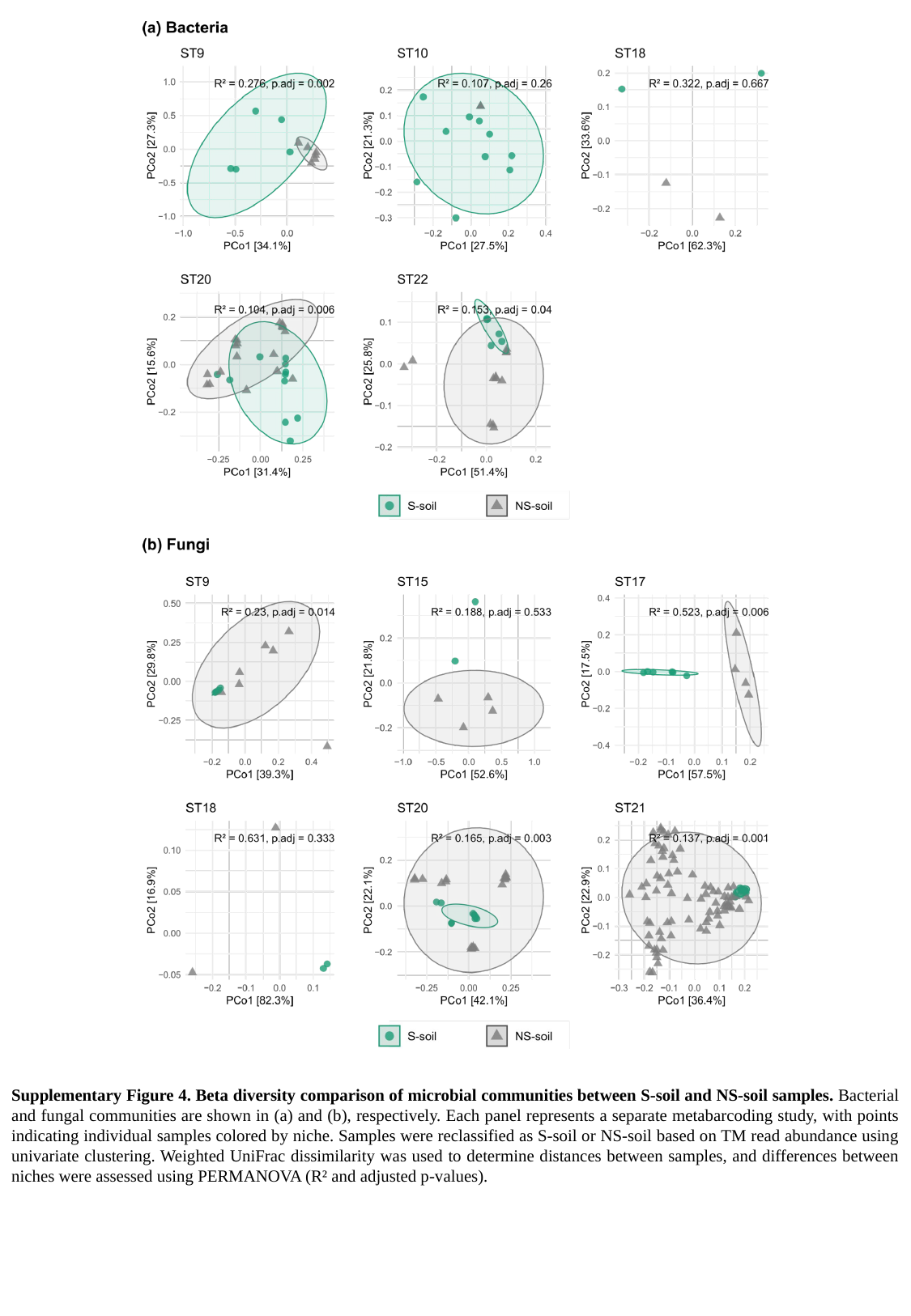

Supplementary Figure 4. Beta diversity comparison of microbial communities between S-soil and NS-soil samples. Bacterial and fungal communities are shown in (a) and (b), respectively. Each panel represents a separate metabarcoding study, with points indicating individual samples colored by niche. Samples were reclassified as S-soil or NS-soil based on TM read abundance using univariate clustering. Weighted UniFrac dissimilarity was used to determine distances between samples, and differences between niches were assessed using PERMANOVA (R² and adjusted p-values).

## Slide 5
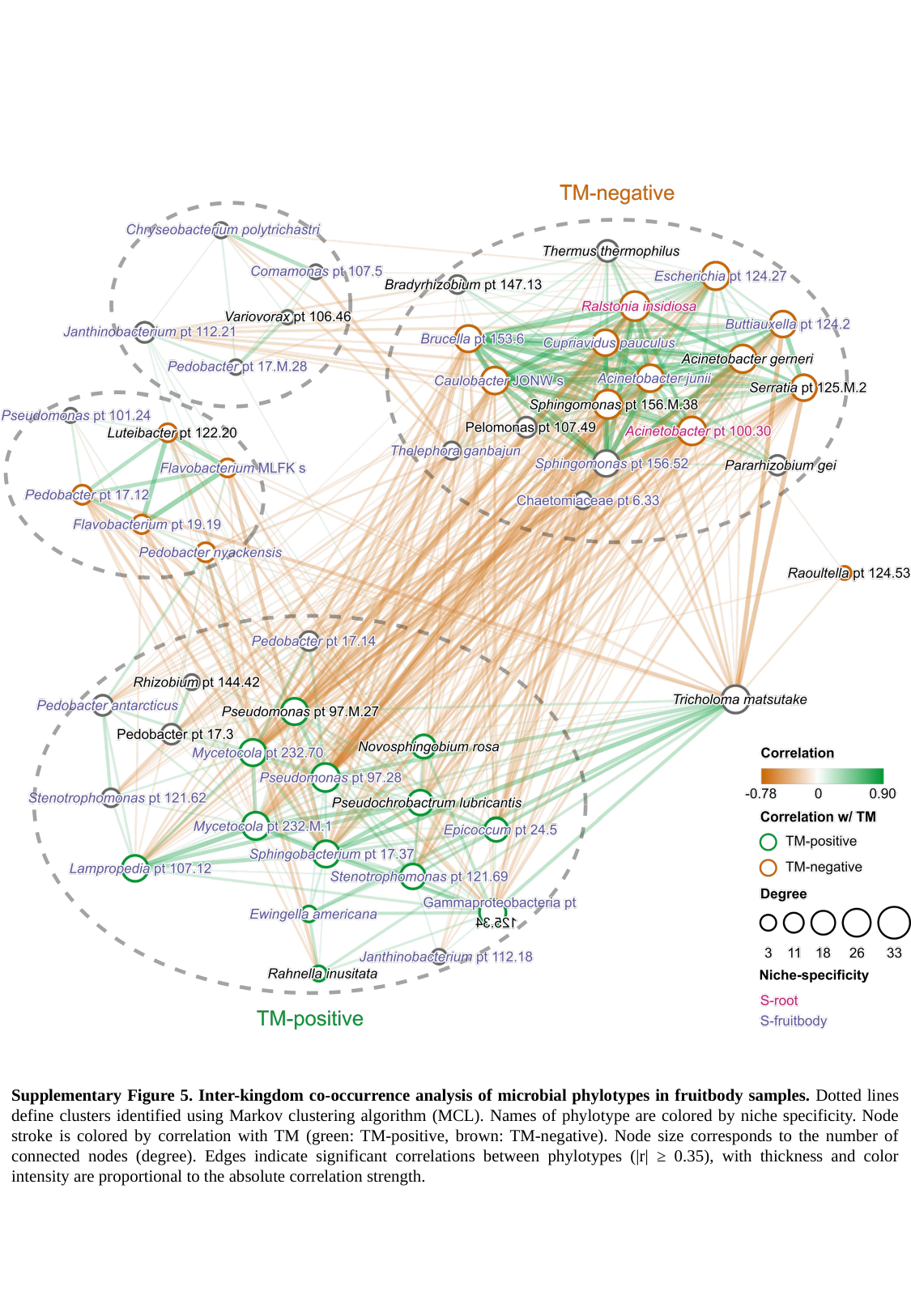

Supplementary Figure 5. Inter-kingdom co-occurrence analysis of microbial phylotypes in fruitbody samples. Dotted lines define clusters identified using Markov clustering algorithm (MCL). Names of phylotype are colored by niche specificity. Node stroke is colored by correlation with TM (green: TM-positive, brown: TM-negative). Node size corresponds to the number of connected nodes (degree). Edges indicate significant correlations between phylotypes (|r| ≥ 0.35), with thickness and color intensity are proportional to the absolute correlation strength.
